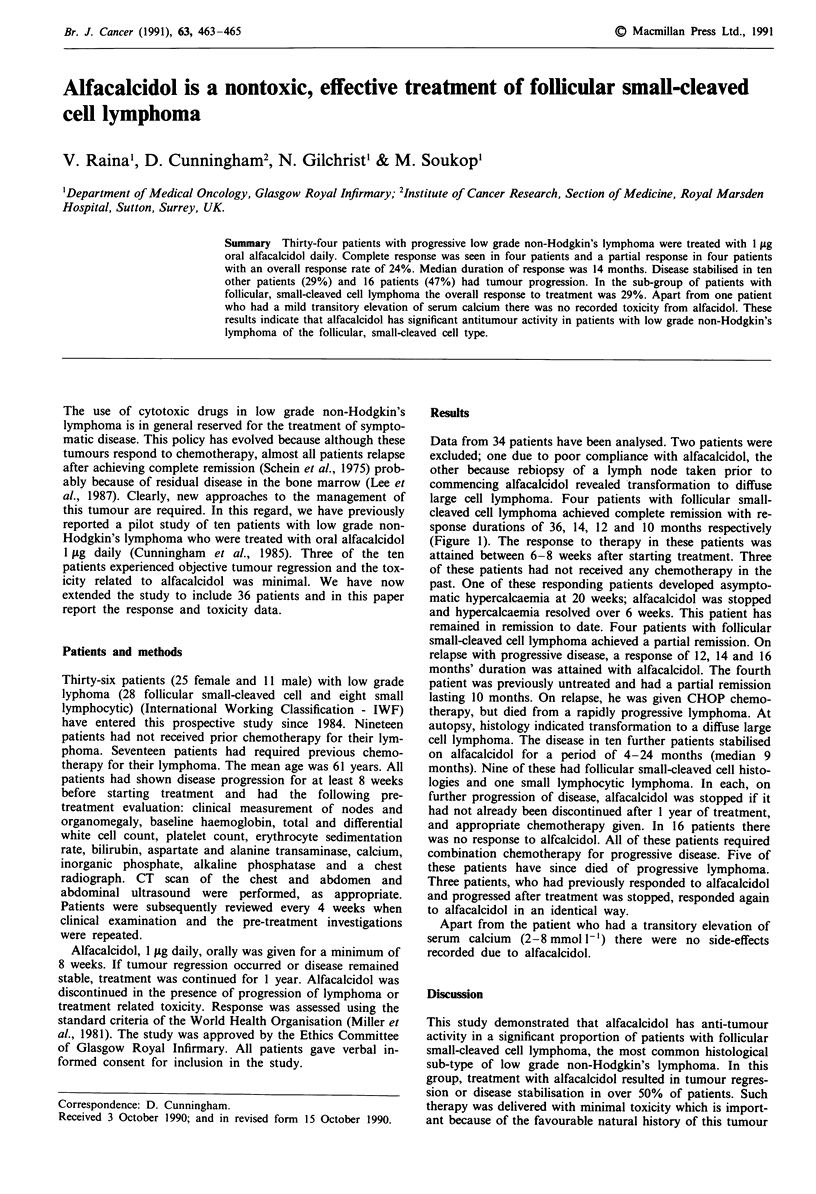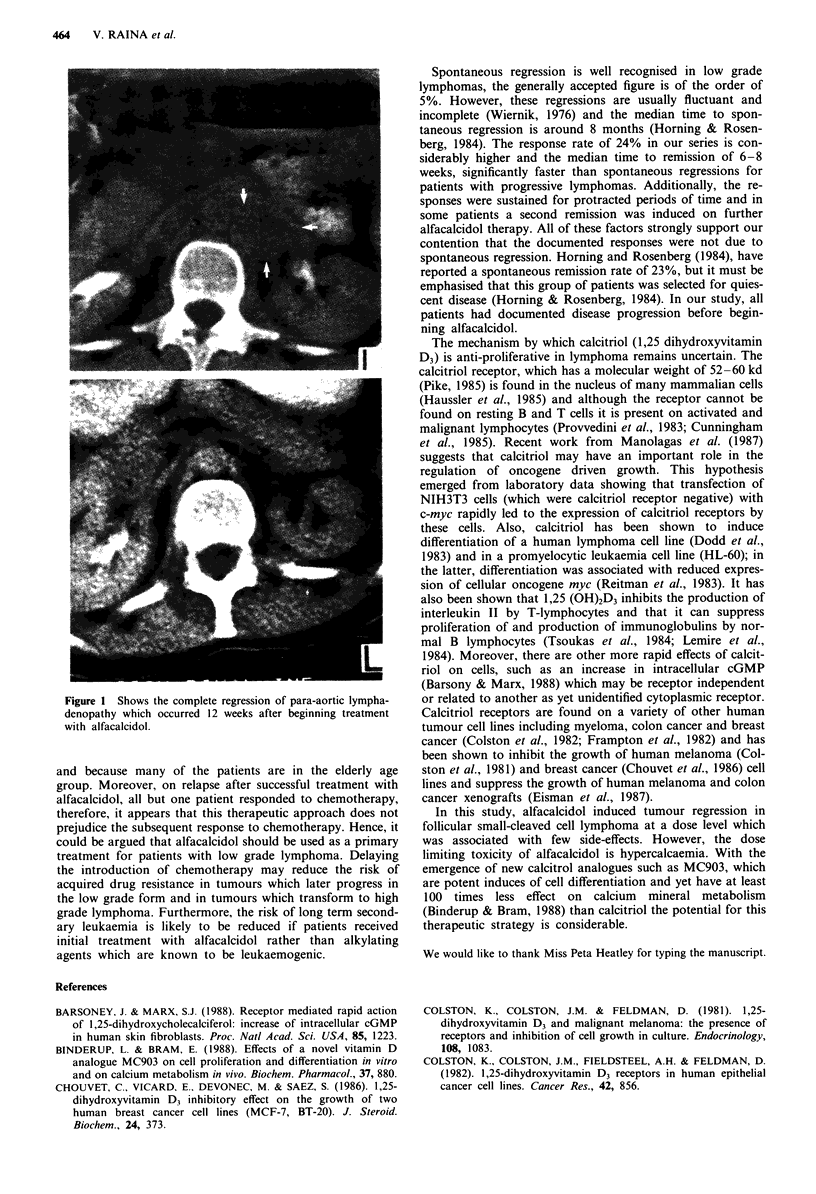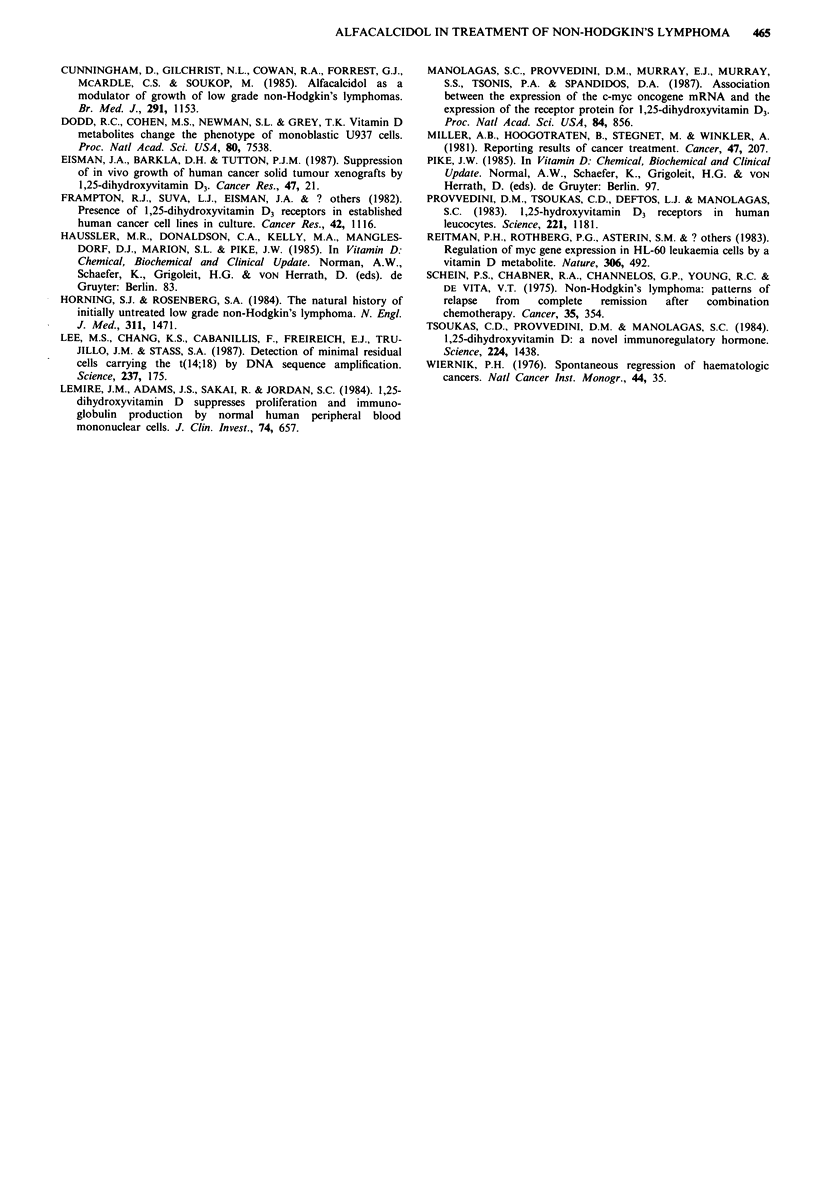# Alfacalcidol is a nontoxic, effective treatment of follicular small-cleaved cell lymphoma.

**DOI:** 10.1038/bjc.1991.108

**Published:** 1991-03

**Authors:** V. Raina, D. Cunningham, N. Gilchrist, M. Soukop

**Affiliations:** Department of Medical Oncology, Glasgow Royal Infirmary, UK.

## Abstract

**Images:**


Br. J. Cancer (1991), 63, 463-465 © Macmillan Press Ltd., 1991

### Alfacalcidol is a nontoxic, effective treatment of follicular small-cleaved cell lymphoma

V. Raina[1], D. Cunningham[2], N. Gilchrist[1] & M. Soukop[1]

[1]*Department of Medical Oncology, Glasgow Royal Infirmary;* [2]*Institute of Cancer Research, Section of Medicine, Royal Marsden Hospital, Sutton, Surrey, UK.*

**Summary** Thirty-four patients with progressive low grade non-Hodgkin's lymphoma were treated with 1 μg oral alfacalcidol daily. Complete response was seen in four patients and a partial response in four patients with an overall response rate of 24%. Median duration of response was 14 months. Disease stabilised in ten other patients (29%) and 16 patients (47%) had tumour progression. In the sub-group of patients with follicular, small-cleaved cell lymphoma the overall response to treatment was 29%. Apart from one patient who had a mild transitory elevation of serum calcium there was no recorded toxicity from alfacidol. These results indicate that alfacalcidol has significant antitumour activity in patients with low grade non-Hodgkin's lymphoma of the follicular, small-cleaved cell type.

The use of cytotoxic drugs in low grade non-Hodgkin's lymphoma is in general reserved for the treatment of symptomatic disease. This policy has evolved because although these tumours respond to chemotherapy, almost all patients relapse after achieving complete remission (Schein *et al.*, 1975) probably because of residual disease in the bone marrow (Lee *et al.*, 1987). Clearly, new approaches to the management of this tumour are required. In this regard, we have previously reported a pilot study of ten patients with low grade non-Hodgkin's lymphoma who were treated with oral alfacalcidol 1 μg daily (Cunningham *et al.*, 1985). Three of the ten patients experienced objective tumour regression and the toxicity related to alfacalcidol was minimal. We have now extended the study to include 36 patients and in this paper report the response and toxicity data.

### Patients and methods

Thirty-six patients (25 female and 11 male) with low grade lyphoma (28 follicular small-cleaved cell and eight small lymphocytic) (International Working Classification - IWF) have entered this prospective study since 1984. Nineteen patients had not received prior chemotherapy for their lymphoma. Seventeen patients had required previous chemotherapy for their lymphoma. The mean age was 61 years. All patients had shown disease progression for at least 8 weeks before starting treatment and had the following pre-treatment evaluation: clinical measurement of nodes and organomegaly, baseline haemoglobin, total and differential white cell count, platelet count, erythrocyte sedimentation rate, bilirubin, aspartate and alanine transaminase, calcium, inorganic phosphate, alkaline phosphatase and a chest radiograph. CT scan of the chest and abdomen and abdominal ultrasound were performed, as appropriate. Patients were subsequently reviewed every 4 weeks when clinical examination and the pre-treatment investigations were repeated.

Alfacalcidol, 1 μg daily, orally was given for a minimum of 8 weeks. If tumour regression occurred or disease remained stable, treatment was continued for 1 year. Alfacalcidol was discontinued in the presence of progression of lymphoma or treatment related toxicity. Response was assessed using the standard criteria of the World Health Organisation (Miller *et al.*, 1981). The study was approved by the Ethics Committee of Glasgow Royal Infirmary. All patients gave verbal informed consent for inclusion in the study.

Correspondence: D. Cunningham.
Received 3 October 1990; and in revised form 15 October 1990.

### Results

Data from 34 patients have been analysed. Two patients were excluded; one due to poor compliance with alfacalcidol, the other because rebiopsy of a lymph node taken prior to commencing alfacalcidol revealed transformation to diffuse large cell lymphoma. Four patients with follicular small-cleaved cell lymphoma achieved complete remission with response durations of 36, 14, 12 and 10 months respectively (Figure 1). The response to therapy in these patients was attained between 6-8 weeks after starting treatment. Three of these patients had not received any chemotherapy in the past. One of these responding patients developed asymptomatic hypercalcaemia at 20 weeks; alfacalcidol was stopped and hypercalcaemia resolved over 6 weeks. This patient has remained in remission to date. Four patients with follicular small-cleaved cell lymphoma achieved a partial remission. On relapse with progressive disease, a response of 12, 14 and 16 months' duration was attained with alfacalcidol. The fourth patient was previously untreated and had a partial remission lasting 10 months. On relapse, he was given CHOP chemotherapy, but died from a rapidly progressive lymphoma. At autopsy, histology indicated transformation to a diffuse large cell lymphoma. The disease in ten further patients stabilised on alfacalcidol for a period of 4-24 months (median 9 months). Nine of these had follicular small-cleaved cell histologies and one small lymphocytic lymphoma. In each, on further progression of disease, alfacalcidol was stopped if it had not already been discontinued after 1 year of treatment, and appropriate chemotherapy given. In 16 patients there was no response to alfcalcidol. All of these patients required combination chemotherapy for progressive disease. Five of these patients have since died of progressive lymphoma. Three patients, who had previously responded to alfacalcidol and progressed after treatment was stopped, responded again to alfacalcidol in an identical way.

Apart from the patient who had a transitory elevation of serum calcium ($2-8\,\mathrm{mmol\,l^{-1}}$) there were no side-effects recorded due to alfacalcidol.

### Discussion

This study demonstrated that alfacalcidol has anti-tumour activity in a significant proportion of patients with follicular small-cleaved cell lymphoma, the most common histological sub-type of low grade non-Hodgkin's lymphoma. In this group, treatment with alfacalcidol resulted in tumour regression or disease stabilisation in over 50% of patients. Such therapy was delivered with minimal toxicity which is important because of the favourable natural history of this tumour

**Figure 1** Shows the complete regression of para-aortic lymphadenopathy which occurred 12 weeks after beginning treatment with alfacalcidol.

and because many of the patients are in the elderly age group. Moreover, on relapse after successful treatment with alfacalcidol, all but one patient responded to chemotherapy, therefore, it appears that this therapeutic approach does not prejudice the subsequent response to chemotherapy. Hence, it could be argued that alfacalcidol should be used as a primary treatment for patients with low grade lymphoma. Delaying the introduction of chemotherapy may reduce the risk of acquired drug resistance in tumours which later progress in the low grade form and in tumours which transform to high grade lymphoma. Furthermore, the risk of long term secondary leukaemia is likely to be reduced if patients received initial treatment with alfacalcidol rather than alkylating agents which are known to be leukaemogenic.

Spontaneous regression is well recognised in low grade lymphomas, the generally accepted figure is of the order of 5%. However, these regressions are usually fluctuant and incomplete (Wiernik, 1976) and the median time to spontaneous regression is around 8 months (Horning & Rosenberg, 1984). The response rate of 24% in our series is considerably higher and the median time to remission of 6–8 weeks, significantly faster than spontaneous regressions for patients with progressive lymphomas. Additionally, the responses were sustained for protracted periods of time and in some patients a second remission was induced on further alfacalcidol therapy. All of these factors strongly support our contention that the documented responses were not due to spontaneous regression. Horning and Rosenberg (1984), have reported a spontaneous remission rate of 23%, but it must be emphasised that this group of patients was selected for quiescent disease (Horning & Rosenberg, 1984). In our study, all patients had documented disease progression before beginning alfacalcidol.

The mechanism by which calcitriol (1,25 dihydroxyvitamin $D_3$) is anti-proliferative in lymphoma remains uncertain. The calcitriol receptor, which has a molecular weight of 52–60 kd (Pike, 1985) is found in the nucleus of many mammalian cells (Haussler *et al.*, 1985) and although the receptor cannot be found on resting B and T cells it is present on activated and malignant lymphocytes (Provvedini *et al.*, 1983; Cunningham *et al.*, 1985). Recent work from Manolagas *et al.* (1987) suggests that calcitriol may have an important role in the regulation of oncogene driven growth. This hypothesis emerged from laboratory data showing that transfection of NIH3T3 cells (which were calcitriol receptor negative) with c-*myc* rapidly led to the expression of calcitriol receptors by these cells. Also, calcitriol has been shown to induce differentiation of a human lymphoma cell line (Dodd *et al.*, 1983) and in a promyelocytic leukaemia cell line (HL-60); in the latter, differentiation was associated with reduced expression of cellular oncogene *myc* (Reitman *et al.*, 1983). It has also been shown that 1,25 $(OH)_2D_3$ inhibits the production of interleukin II by T-lymphocytes and that it can suppress proliferation of and production of immunoglobulins by normal B lymphocytes (Tsoukas *et al.*, 1984; Lemire *et al.*, 1984). Moreover, there are other more rapid effects of calcitriol on cells, such as an increase in intracellular cGMP (Barsony & Marx, 1988) which may be receptor independent or related to another as yet unidentified cytoplasmic receptor. Calcitriol receptors are found on a variety of other human tumour cell lines including myeloma, colon cancer and breast cancer (Colston *et al.*, 1982; Frampton *et al.*, 1982) and has been shown to inhibit the growth of human melanoma (Colston *et al.*, 1981) and breast cancer (Chouvet *et al.*, 1986) cell lines and suppress the growth of human melanoma and colon cancer xenografts (Eisman *et al.*, 1987).

In this study, alfacalcidol induced tumour regression in follicular small-cleaved cell lymphoma at a dose level which was associated with few side-effects. However, the dose limiting toxicity of alfacalcidol is hypercalcaemia. With the emergence of new calcitrol analogues such as MC903, which are potent induces of cell differentiation and yet have at least 100 times less effect on calcium mineral metabolism (Binderup & Bram, 1988) than calcitriol the potential for this therapeutic strategy is considerable.

We would like to thank Miss Peta Heatley for typing the manuscript.

### References

BARSONEY, J. & MARX, S.J. (1988). Receptor mediated rapid action of 1,25-dihydroxycholecalciferol: increase of intracellular cGMP in human skin fibroblasts. *Proc. Natl Acad. Sci. USA*, **85,** 1223.

BINDERUP, L. & BRAM, E. (1988). Effects of a novel vitamin D analogue MC903 on cell proliferation and differentiation *in vitro* and on calcium metabolism *in vivo*. *Biochem. Pharmacol.*, **37,** 880.

CHOUVET, C., VICARD, E., DEVONEC, M. & SAEZ, S. (1986). 1,25-dihydroxyvitamin $D_3$ inhibitory effect on the growth of two human breast cancer cell lines (MCF-7, BT-20). *J. Steroid. Biochem.*, **24,** 373.

COLSTON, K., COLSTON, J.M. & FELDMAN, D. (1981). 1,25-dihydroxyvitamin $D_3$ and malignant melanoma: the presence of receptors and inhibition of cell growth in culture. *Endocrinology*, **108,** 1083.

COLSTON, K., COLSTON, J.M., FIELDSTEEL, A.H. & FELDMAN, D. (1982). 1,25-dihydroxyvitamin $D_3$ receptors in human epithelial cancer cell lines. *Cancer Res.*, **42,** 856.

CUNNINGHAM, D., GILCHRIST, N.L., COWAN, R.A., FORREST, G.J., MCARDLE, C.S. & SOUKOP, M. (1985). Alfacalcidol as a modulator of growth of low grade non-Hodgkin's lymphomas. *Br. Med. J.*, **291**, 1153.

DODD, R.C., COHEN, M.S., NEWMAN, S.L. & GREY, T.K. Vitamin D metabolites change the phenotype of monoblastic U937 cells. *Proc. Natl Acad. Sci. USA*, **80**, 7538.

EISMAN, J.A., BARKLA, D.H. & TUTTON, P.J.M. (1987). Suppression of in vivo growth of human cancer solid tumour xenografts by 1,25-dihydroxyvitamin $D_3$. *Cancer Res.*, **47**, 21.

FRAMPTON, R.J., SUVA, L.J., EISMAN, J.A. & ? others (1982). Presence of 1,25-dihydroxyvitamin $D_3$ receptors in established human cancer cell lines in culture. *Cancer Res.*, **42**, 1116.

HAUSSLER, M.R., DONALDSON, C.A., KELLY, M.A., MANGLESDORF, D.J., MARION, S.L. & PIKE, J.W. (1985). In *Vitamin D: Chemical, Biochemical and Clinical Update*. Norman, A.W., Schaefer, K., Grigoleit, H.G. & VON Herrath, D. (eds). de Gruyter: Berlin. 83.

HORNING, S.J. & ROSENBERG, S.A. (1984). The natural history of initially untreated low grade non-Hodgkin's lymphoma. *N. Engl. J. Med.*, **311**, 1471.

LEE, M.S., CHANG, K.S., CABANILLIS, F., FREIREICH, E.J., TRUJILLO, J.M. & STASS, S.A. (1987). Detection of minimal residual cells carrying the t(14;18) by DNA sequence amplification. *Science*, **237**, 175.

LEMIRE, J.M., ADAMS, J.S., SAKAI, R. & JORDAN, S.C. (1984). 1,25-dihydroxyvitamin D suppresses proliferation and immunoglobulin production by normal human peripheral blood mononuclear cells. *J. Clin. Invest.*, **74**, 657.

MANOLAGAS, S.C., PROVVEDINI, D.M., MURRAY, E.J., MURRAY, S.S., TSONIS, P.A. & SPANDIDOS, D.A. (1987). Association between the expression of the c-myc oncogene mRNA and the expression of the receptor protein for 1,25-dihydroxyvitamin $D_3$. *Proc. Natl Acad. Sci. USA*, **84**, 856.

MILLER, A.B., HOOGOTRATEN, B., STEGNET, M. & WINKLER, A. (1981). Reporting results of cancer treatment. *Cancer*, **47**, 207.

PIKE, J.W. (1985). In *Vitamin D: Chemical, Biochemical and Clinical Update*. Normal, A.W., Schaefer, K., Grigoleit, H.G. & VON Herrath, D. (eds). de Gruyter: Berlin. 97.

PROVVEDINI, D.M., TSOUKAS, C.D., DEFTOS, L.J. & MANOLAGAS, S.C. (1983). 1,25-hydroxyvitamin $D_3$ receptors in human leucocytes. *Science*, **221**, 1181.

REITMAN, P.H., ROTHBERG, P.G., ASTERIN, S.M. & ? others (1983). Regulation of myc gene expression in HL-60 leukaemia cells by a vitamin D metabolite. *Nature*, **306**, 492.

SCHEIN, P.S., CHABNER, R.A., CHANNELOS, G.P., YOUNG, R.C. & DE VITA, V.T. (1975). Non-Hodgkin's lymphoma: patterns of relapse from complete remission after combination chemotherapy. *Cancer*, **35**, 354.

TSOUKAS, C.D., PROVVEDINI, D.M. & MANOLAGAS, S.C. (1984). 1,25-dihydroxyvitamin D: a novel immunoregulatory hormone. *Science*, **224**, 1438.

WIERNIK, P.H. (1976). Spontaneous regression of haematologic cancers. *Natl Cancer Inst. Monogr.*, **44**, 35.